# Molecular characterisation of tick-borne pathogens in cattle in kenya: insights from blood, ticks, and skin swab analyses

**DOI:** 10.1186/s12917-025-05014-1

**Published:** 2025-10-02

**Authors:** Dennis Getange, Samson Mukaratirwa, Joel L. Bargul, Rua Khogali, John Ng’iela, James Kabii, Daniel K. Masiga, Jandouwe Villinger

**Affiliations:** 1 International Centre of Insect Physiology and Ecology (icipe), P.O Box 30772-00100, Nairobi, Kenya; 2 School of Life Sciences, University of KwaZulu-Natal, Westville Campus, Private Bag X54001, Durban, 4000 South Africa; 3 One Health Centre for Zoonoses and Tropical Veterinary Medicine, Ross University School of veterinary Medicine, Basseterre, West Indies St Kitts and Nevis; 4 Department of Biochemistry, Jomo Kenyatta University of Agriculture and Technology, P.O Box 62000-00200, Nairobi, Kenya; 5 Department of Zoology and Entomology, University of Pretoria, Private Bag 20, Pretoria, 0028 South Africa

**Keywords:** Ticks, Tick-borne pathogens, *Amblyomma*, Q fever, *Anaplasma*, *Ehrlichia*, *Rickettsia*, *Theileria*

## Abstract

**Background:**

Ticks pose a major threat to livestock and human health in sub-Saharan Africa, with climate change and pastoral movements fueling their spread. Few studies have integrated multiple sample types to characterize tick-borne pathogens (TBPs) in cattle in Kenya. This knowledge gap hinders the development of effective surveillance and control strategies, leaving vulnerable populations and their livestock susceptible to these persistent threats.

**Methods:**

We screened 280 bovine blood samples, 589 tick pools, and 284 non-invasive skin swabs from cattle in northern (Marsabit) and southern (Kajiado) Kenya by high-resolution melting analysis and Sanger sequencing of PCR products.

**Results:**

*Rhipicephalus* spp. (47.1%), *Hyalomma* spp. (30.8%), and *Amblyomma* spp. (22.1%) were prevalent, with *Rhipicephalus evertsi* only found in Kajiado and *Rhipicephalus camicasi* in Marsabit. In blood, *Anaplasma* spp. (62.9%; *A. marginale*, *A. platys*, *A. ovis*) and *Theileria* spp. (34.6%; *T. velifera*, *T. mutans*) were dominant. Tick pools harbored *Coxiella burnetii*, *Rickettsia africae*, *Rickettsia aeschlimannii*, *Anaplasma marginale*, *Theileria velifera*, *T. ovis*, and *Babesia occultans*, and for the first time two co-circulating *Ehrlichia ruminantium* strains (Welgevonden and Kumm2). Notably, *C. burnetii* and *T. ovis* were detected only in Marsabit, and *T. mutans* only in Kajiado. Skin swabs from tick predilection sites (ears, anal region) yielded *R. africae*, *R. aeschlimannii*, and *T. velifera* at low positivity, while nose swabs were negative.

**Conclusions:**

Detection of zoonotic pathogens such as *C. burnetii* and *R. africae* underscores critical public health risks, and co-infections in cattle reinforce the need for robust, integrated surveillance. Although skin swabs demonstrated limited diagnostic yield, they remain a promising non-invasive sampling approach. These findings highlight the value of targeted acarological research and coordinated control programs under a One Health framework.

## Introduction

Over the past two decades, the incidence of tick-borne diseases caused by bacterial, protozoan, and viral pathogens has steadily increased [1]. Worldwide, changes such as climate change, habitat fragmentation, and human activities have contributed to the spread of ticks and the emergence and re-emergence of tick-borne pathogens (TBPs) [2, 3]. Interactions between humans, domestic animals, and wildlife create pathways for ectoparasite transmission, particularly ticks, which can facilitate the emergence and spread of zoonotic diseases. This interconnectedness aligns with the World Health Organization’s One Health concept, which recognizes that human health is intrinsically linked to animal health and environmental conditions [4]. Globally, an estimated 61% of human diseases have been documented to have a zoonotic origin, with approximately 75% of these re-emerging zoonotic infections originating from wildlife species [5]. These include a wide variety of pathogens, which cause debilitating and sometimes fatal illnesses in both humans and animals [6, 7].

In Kenya and other sub-Saharan Africa (SSA) countries where communities rely heavily on livestock production as a source of their livelihood [8], tick-borne diseases (TBDs) like theileriosis, ehrlichiosis, babesiosis, and anaplasmosis transmitted by ticks are the leading causes of livestock losses through morbidity, mortality, and productivity losses [9, 10]. The predominantly warm and, in some areas, seasonally humid climate of SSA fosters widespread tick proliferation, facilitating the transmission of pathogens to both animals and humans [11]. Studies in Kenya have reported exposure of cattle to bacterial (*Ehrlichia*, *Anaplasma*, *Rickettsia*, *Coxiella burnetii*), protozoal (*Theileria*, *Babesia*) [12–15] and viral (Crimean-Congo haemorrhagic fever virus (CCHF), *Phlebovirus*) TBPs [14, 16, 17]. The unrestricted movement of livestock, through nomadic pastoralism or cross-border trade, further spreads infection to naïve herds and endangers livestock handlers, veterinarians, abattoir workers, and surrounding communities [18]. However, while numerous studies have documented TBDs in sub-Saharan Africa, many rely on a single sample type—typically blood or ticks—thus overlooking alternate sampling methods like skin swabs that may detect additional pathogens or provide more practical, non-invasive surveillance options. Moreover, most surveys concentrate on specific regions, leaving major gaps in our understanding of TBPs across diverse agro-ecological zones within Kenya. Despite the high disease burden, there remains a paucity of robust data on the diversity and molecular epidemiology of TBPs across the country’s varied agro-ecological zones, highlighting the need for targeted surveillance in Kenya. Adopting an intensive, continuous screening strategy under a One Health approach is therefore critical for the early detection of TBPs, allowing for prevention of significant economic losses in livestock production.

Skin swabs present a potentially valuable non-invasive method for TBP surveillance, offering a complementary approach to the gold standard of blood and tick sampling. This method is particularly relevant in pastoral communities where traditional sampling can be logistically difficult and stressful for livestock. It is important to note, however, that the presence of TBP DNA on skin swabs may be the result of contamination from tick feces or other environmental sources, not necessarily an active infection in the host. While acknowledging the potential for lower detection rates, skin swabs could serve as a practical, field-friendly tool for preliminary screening or when conventional methods are not a viable option.

In this study, we aimed to determine the (i) prevalence and molecular diversity of economically important TBPs in ticks and blood from cattle in two arid and semi-arid livestock-keeping regions of Kenya (Marsabit County in the north and Kajiado County in the south), and (ii) feasibility of using non-invasive skin swab samples from tick-bite sites for surveillance of TBPs in cattle. By comparing pathogen detection rates from blood, tick, and skin swab samples, our findings could offer insights into the epidemiology of TBPs in arid and semi-arid rangelands, while also informing the design of integrated tick control programs. Ultimately, these results aim to support strategies that can reduce disease burden, enhance livestock productivity, and mitigate the public health risks associated with tick-borne infections, priorities that align with the One Health vision for sub-Saharan Africa.

## Materials and methods

### Study area

**Fig. 1:**
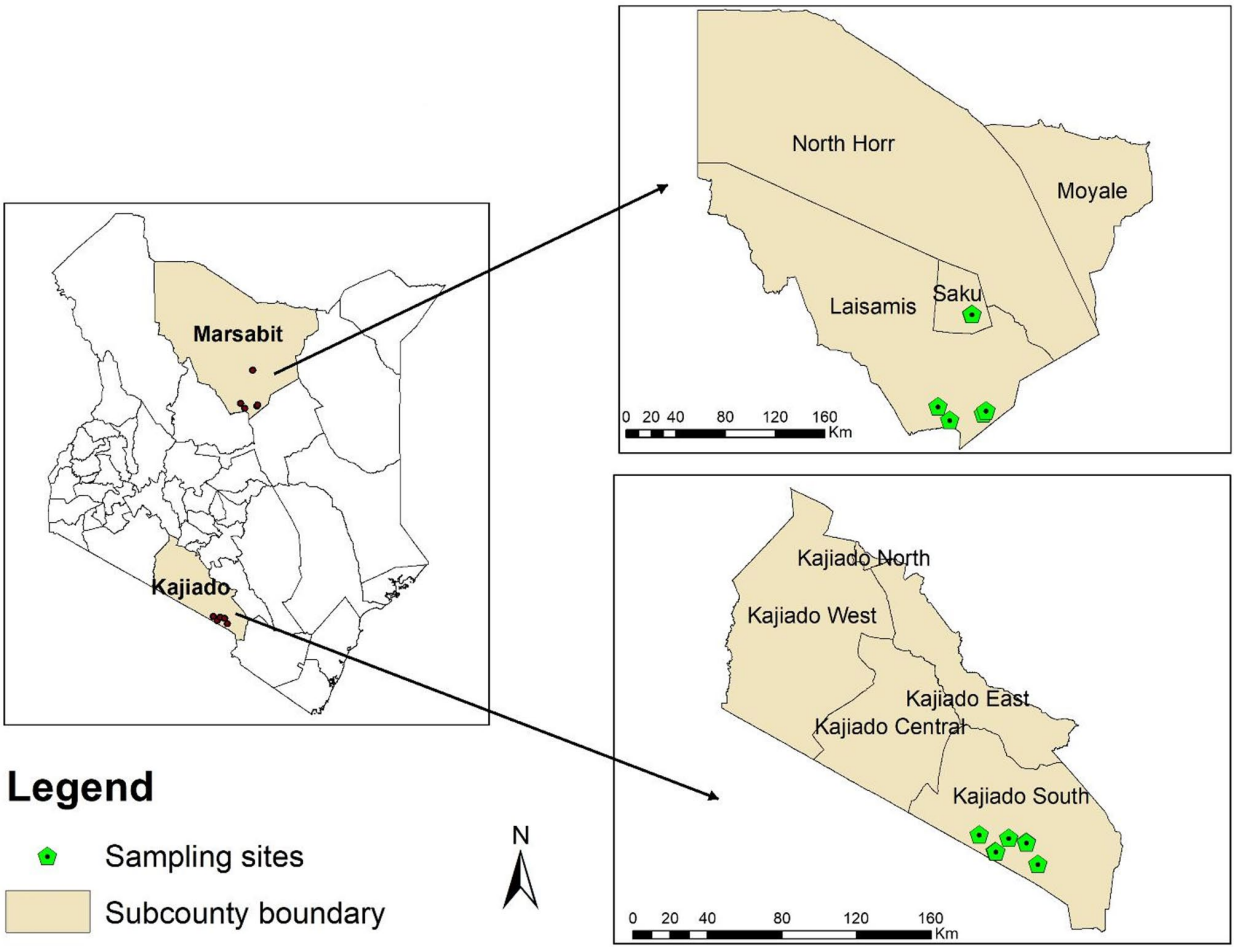
A map of Kenya showing the sampling sites in Marsabit and Kajiado counties. The maps were created using the open-source software, QGIS v.3.14.16

This study was conducted in Marsabit and Kajiado counties (Fig. 1) in November 2022 and April 2024. Marsabit County, a vast region in northern Kenya with an area of 70,961 km^2^ and shares a border with Ethiopia, covers over 15% of the Kenya’s total land area. The landscape is characterized by a unique landscape, encompassing both arid and semi-arid ecosystems [19]. On the other hand, Kajiado County with an area of 21,900.9 km^2^, located in southern Kenya and shares a border with Tanzania, is characterized by rolling hills and open savannas. The landscape of Kajiado is largely dominated by grasslands and acacia woodlands, which support an array of wildlife [20]. Both counties face challenges such as climate change, habitat fragmentation, and human-wildlife conflict, which require careful management and conservation efforts to preserve their natural heritage. Ticks, blood, and skin swabs were collected from cattle in Thurusi in Laisamis sub-county and Badassa in Saku sub-county in Marsabit County and Kimana in Kajiado South sub-county in Kajiado County (Fig. 1).

### Collection of ticks, blood, and skin swab samples

This cross-sectional field study utilized an opportunistic sampling approach, collecting ticks, blood, and skin swabs from 280 cattle in Marsabit (*n* = 211) and Kajiado (*n* = 69) counties between November 2022 and April 2024. Baseline data was collected and recorded in relevant forms, and this included information on the sampling location, unique animals ID, sex, age, recent health history, and the status about tick infestation before sample collection. Thereafter, whole blood (4 mL) was drawn from the jugular vein of each individual cattle using 18-gauge vacutainer BD needle and placed into sterile 4-mL EDTA vacutainer tubes (BD Vacutainer^®^). Blood samples in the EDTA vacutainers were transferred into well labelled-cryovials and preserved in liquid nitrogen awaiting transportation. Skin swab samples were collected by gently rubbing a sterile cotton-tipped swab over the skin surface of each animal, focusing on areas with visible ectoparasites or lesions. The swabs were placed in sterile cryovials and labelled with unique animal identifier and the site where they collected from. Live ticks were manually removed from visible predilection sites (head, ears, neck, dewlap, belly, legs, tail, and udder in the case of females and testes in males, and tail) from cattle using a pair of serrated forceps and placed in 15-mL sterile falcon tubes containing moistened cotton wool to maintain humidity. The tick samples were then labelled with the corresponding animal identification number, location, and date of collection. The Falcon tubes were then placed in a portable cooler box with ice packs to maintain the appropriate relative humidity and ambient temperature. All samples collected in this study were transported to International Centre of Insect Physiology and Ecology (*icipe*) Martin Lüscher Emerging Infectious Diseases (ML-EID) Laboratory in Nairobi for further analysis.

### Morphological identification of ticks

Adult ticks collected from cattle were morphologically identified to species level using established taxonomic keys [21–23] under a Stemi 2000-C light microscope (Zeiss, Oberkochen, Germany) and photographed with an Axio-cam ERc 5 s camera (Zeiss) mounted on the microscope. Anatomical features were used to differentiate between male and female ticks. Excessively engorged ticks were removed during tick identification and excluded from subsequent analysis to minimise vertebrate host DNA in nucleic acid extractions. After identification, adult ticks were pooled into groups of one to eight individuals based on tick species, sex, sampling site, and date of collection.

### DNA extraction from ticks, blood, and skin swabs

The ticks were washed in 1% bleach for 30 s, 70% ethanol for 30 s and rinsed in nuclease free water for 30 s to remove external contaminants. The ticks were then frozen in liquid nitrogen before homogenising them in 1.5-mL microfuge tubes containing 150 mg of 0.1-mm and 750 mg of 2.0-mm yttria-stabilised zirconium (YSZ) oxide beads (Glen Mills, Clifton, NJ, USA) using a Mini-Beadbeater-16 (BioSpec, Bartlesville, OK, USA) for 1 min. Genomic DNA was extracted from the homogenized ticks, blood, and skin swabs using ISOLATE II Genomic DNA Kit (Bioline Meridian, Inc., UK) according to the manufacturer’s instructions. The DNA was eluted into 80 µL TE buffer and the purity assessed using a Eppendorf BioSpectrometer (Humburg, Germany), yielding an A260/A280 ratio between 1.7 and 1.9. It was then stored at −20 °C before PCR amplification.

### Molecular detection of TBPs in ticks, blood, and skin swab samples using PCR-HRM

Five hundred and eighty-nine tick pools, 280 blood samples, and 284 skin swabs DNA extracts were screened for TBPs of genera *Coxiella burnetii*, *Anaplasma*, *Ehrlichia*, *Rickettsia*, *Babesia*, and *Theileria* by high-resolution melting (HRM) analysis in Mic PCR Cyclers (Bio Molecular Systems, Upper Coomera, Queensland, Australia) using published primers [12, 24] (Table 1). The primer pairs (Table 1) were used to amplify 16 S rRNA gene fragments of *Anaplasma* (Anaplasma JVF and JVR), *Ehrlichia* (Ehrlichia 16S F and R), and *Rickettsia* spp. (Rick-F and R). and 18S rRNA gene of *Babesia* and *Theileria* spp. (RLB F and R). We also screened for *C. burnetii* using *C._burnetii*_HRM-F and *C._burnetii*_HRM-R primers [25]. The PCR reaction mixture consisted of 0.5 µL of 10 pmol/µL of each forward and reverse primer, 2 µL of FIREPol^®^ EvaGreen^®^ HRM mix (Solis Biodyne, Tartu, Estonia), and 1 µL of genomic DNA as template, with the volume adjusted to 10 µL using nuclease-free water. The amplification conditions were performed as described by Mwamuye et al. [12], Omondi et al. [26], Getange et al. [24] for *Anaplasma*, *Ehrlichia*, *Rickettsia*, *Theileria*, and *Babesia* and Kamau et al. [25] for *C. burnetii*.

**Table 1:** Primers used for molecular detection of TBPs in ticks, blood and skin swabs

Primer name	Gene target	Target size (bp)	Sequence (5’–3’)	Primer reference
Rick-F Rick-R	*Rickettsia* 16 S rRNA	364	GAACGCTATCGGTATGCTTAACACA CATCACTCACTCGGTATTGCTGGA	[27]
120–2788 120–3599	*Rickettsia* ompB	856	AAACAATAATCAAGGTACTGT TACTTCCGGTTACAGCAAAGT	[28]
C_burnetii_HRM-F C_burnetii_HRM-R	*Coxiella* IS1111	150	GGAACTTGTCAGAGATGATTTGGT AGAGTTCCCGACTTGACTCG	[25]
Trans1 Trans2	*Coxiella* IS1111	687	TGGTATTCTTGCCGATGAC GATCGTAACTGCTTAATAAACCG	[29]
Ehrlichia16S F Ehrlichia16S R	*Ehrlichia* 16 S rRNA	200	CGTAAAGGGCACGTAGGTGGACTA CACCTCAGTGTCAGTATCGAACCA	[30]
PER1 PER2	*Ehrlichia* 16 S rRNA	451	TTTATCGCTATTAGATGAGCCTATG CTCTACACTAGGAATTCCGCTAT	[31]
EHR16SD 1492R	*Anaplasma*/*Ehrlichia* 16 S rRNA	1030	GGTACCYACAGAAGAAGTCC GGTTACCTTGTTACGACTT	[32, 33]
AnaplasmaJVF AnaplasmaJVR	*Anaplasma* 16 S rRNA	300	CGGTGGAGCATGTGGTTTAATTC CGRCGTTGCAACCTATTGTAGTC	[12]
RLB F RLB R	*Theileria*/*Babesia* 18 S rRNA	460–520	GAGGTAGTGACAAGAAATAACAATA TCTTCGATCCCCTAACTTTC	[34]

### Identification of pathogen PCR-HRM profiles through DNA sequencing

Representatives of samples with unique HRM profiles of *Anaplasma* spp. were re-amplified using primer sets EHR16SD-1492R and *Ehrlichia* spp. using PER1-PER2. *Rickettsia* spp. was reamplified using 120–2788 and 120–3599 and *C. burnetii* using Trans 1 and Trans 2 primer (Table 1). These PCRs were performed in a ProFlex PCR Systems thermocycler (Applied Biosystems, Foster City, CA, USA) in a 15-µL final reaction volume consisting of 3 µL of 5x HOT FIREPol^®^ Blend Master Mix (Solis BioDyne, Tartu, Estonia), 1.5 µL of the template and 0.75 µl each of 10 pmol/µL forward and reverse primers. Previously described cycling conditions were used [24, 25, 35]. Successful amplification was determined by resolving 5 µL of PCR amplicons in ethidium bromide-stained 2% gel electrophoresis and visualized under ultraviolet light. The remaining PCR amplicons were purified using ExoSAP (New England Biolabs, UK) based on the manufacturer’s instructions (New England Biolabs, Frankfurt, Germany) and outsourced for sanger sequencing (Macrogen, The Netherlands). All study sequences were deposited in the GenBank of NCBI nucleotide database.

### Phylogenetic and statistical analysis

The resulting forward and reverse pathogen sequence chromatograms were edited, trimmed and aligned using Geneious Prime software version 2024.0.7 [36] to generate consensus sequences, which were queried against known annotated sequences in the GenBank database (http://www.ncbi.nlm.nih.gov) using BLAST nucleotide search tool [37] to confirm their identity and relation to the homologous sequences of reference. Maximum-likelihood phylogenies were constructed and inferred for each gene using PhyML v. 3.0 with automatic model selection based on Akaike information criterion. Trees were visualised in FigTree 1.4.4 [38]. Infection rates were calculated as a percentage of infected bovine blood/tick/skin swabs relative to the total number of screened bovine blood/tick/skin swabs. The UpSetR diagrams were generated using UpSetR package in R statistical software version 4.2.1 [39].

## Results

### Tick infestation

A total of 2,747 partially engorged ticks were collected from 280 cattle from Marsabit and Kajiado counties. Most of the assessed cattle were infested by *Rh. pulchellus* (46.1%), followed by *Hyalomma rufipes* (27.4%), and *Amblyomma gemma* (20.8%). Other tick species found on cattle included *Hyalomma impeltatum* (2.6%), *Hyalomma truncatum* (0.7%), *Rhipicephalus evertsi* (0.5%), *Rhipicephalus camicasi* (0.1%). In addition to *Rhipicephalus evertsi* and *Rhipicephalus camicasi*, which were absent in Marsabit and Kajiado counties, respectively, other tick species were found in both counties (Table 2).

**Table 2:** Distribution of cattle tick species in Marsabit and Kajiado counties, Kenya

Prevalence						
Tick species	**Marsabit**			**Kajiado**		
	**Sex**			**Sex**		
	**Male**	**Female**	**No. of ticks (%)**	**Male**	**Female**	**No. of ticks (%)**
*Amblyomma gemma*	291	138	429 (19.54%)	132	47	179 (30.30%)
*Hyalomma rufipes*	479	226	705 (32.11%)	41	8	49 (8.26%)
*Hy. truncatum*	12	4	16 (0.73%)	2	2	4 (0.85%)
*Hy. impeltatum*	45	25	70 (3.19%)	0	2	2 (0.21%)
*Rhipicephalus* sp.	0	0	0 (0%)	3	3	6 (1.27%)
*Rh. pulchellus*	537	436	973 (44.33%)	152	140	292 (56.14%)
*Rh. evertsi*			0 (0%)	14	6	20 (2.97%)
*Rh. camicasi*	2	0	2 (0.09%)	0	0	0 (0)

### Tick-borne pathogens in cattle blood

**Fig. 2:**
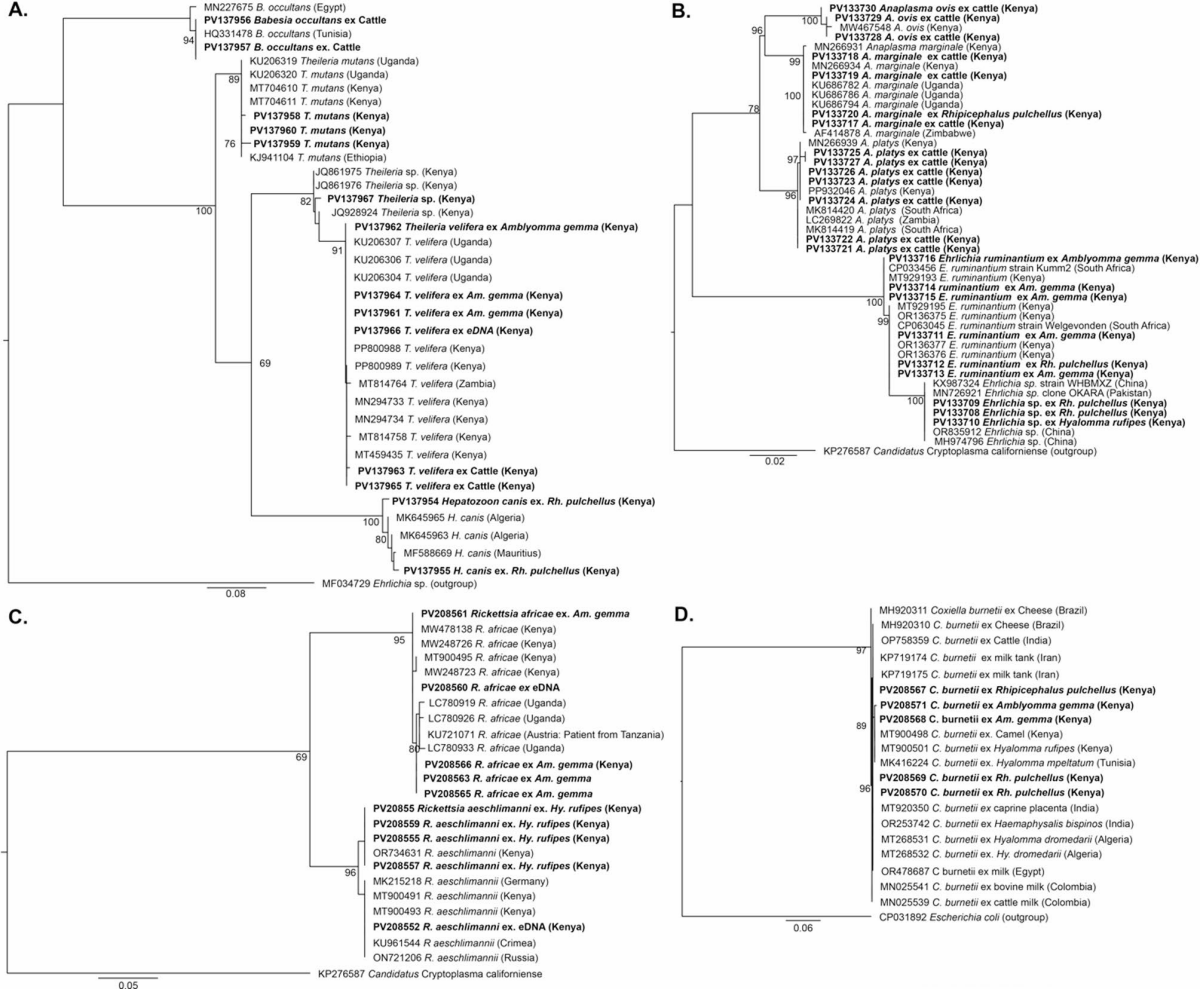
Maximum likelihood phylogenies of (**A**) *Rickettsia* spp. *ompB* gene detected in ticks and skin swabs, and (**B**) *Anaplasma* spp. 16S rRNA gene (**C**) *Theileria* spp. 18S rRNA gene, and (**D**) *C. burnetii* IS1111 gene from cattle in Marsabit County and Kajiado County Kenya. Sequences from this study are in bold

Out of the 280 (211 in Marsabit and 69 in Kajiado) bovine blood DNA screened for the presence of TBPs, we detected *Anaplasma* spp. (62.9%), *Theileria* spp. (35.9%) and *Babesia* spp. (1.4%) by PCR-HRM and confirmed through gene sequencing. As there were distinct HRM profiles for *Anaplasma* amplicons, all *Anaplasma* spp., upon sequencing, were further identified as *A. marginale*, *A. platys*, and *A. ovis*. Three *Anaplasma* sequences (PV133717-PV133719) shared 100% identity with *A. marginale* from Kenya (PP800988 and MT459435) and Uganda (KU206304-7). Three *Anaplasma* sequences (PV133728-PV133730) shared 100% similarity with *A. ovis* from Kenya (MW467548), and seven sequences (PV133721-PV133727) shared 100% similarity with *(A) platys* like sequences from Kenya (PP932046 and MN266939) and South Africa (MK814420). *Theileria* spp. were confirmed as *T. velifera* and *T. mutans*. *Theileria* spp. sequences (PV137963, PV137965) shared 100% similarity with *T. velifera* from Kenya (PP800988 and MT459435) and Uganda (KU206304-7) and three sequences (PV137958-PV137960) shared 99–100% similarity with reference *T. mutans* sequences from Kenya (MT704610-11) and Ethiopia (KJ941104). *Babesia* spp. were confirmed as *(B) occultans*. Two *B. occultans*. sequences (PV137956-PV137957) shared 100% similarity with *B. occultans* from Egypt (MN227675) (Fig. 2; Table 4). We did not detect *Ehrlichia* spp., *Rickettsia* spp., or *Coxiella burnetii* DNA in bovine blood.

### Tick-borne pathogens in ticks

We detected *Anaplasma*, *Ehrlichia*, *Rickettsia*, *Theileria*, *Hepatozoon*, and *Coxiella burnetii* in tick pools (Fig. 2, Fig. 4; Table 3). *Coxiella burnetii* (PV208567 – PV208571), which shared 99–100% similarity with sequences from Kenya (MT900498 and MT900501) and Tunisia (MK416224) was detected in *Am. gemma*, *Hy. rufipes*, and *Rh. pulchellus* tick pools. However, *C. burnetii* DNA was reported in a much higher percentage in *Rh. pulchellus* and *Am. gemma* than in *Hy. rufipes*, and only in Marsabit, not in Kajiado (Table 3).

**Table 3:** Pool positivity rates for tick-borne pathogens detected in ticks collected from Marsabit and Kajiado counties

No. of positive pools (pool positivity rates %)									
Location	Tick species	*Am. gemma*	*Hy. rufipes*	*Hy. truncatum*	*Hy. impeltatum*	*Rh. pulchellus*	*Rh. camicasi*	*Rh. evertsi*	*Rhipicephalus *sp.
Marsabit County	No. of tick pools	114	193	8	20	153	2		
	*Coxiella burnetii*	29 (25.4)	1 (0.5)			36 (23.5)			
	*Anaplasma marginale*	2 (1.8)	2 (1.0)			5 (3.2)			
	*Theileria ovis*		1 (0.5)			1 (0.7)			
	*T. velifera*	6 (5.3)							
	*Hepatozoon canis*					1 (0.7)			
	*Rickettsia africae*	68 (59.6)				38 (24.8)			
	*R. aeschlimannii*		98 (50.8)	4 (50.0)	7 (35.0)				
	*Ehrlichia* sp.		50 (25.9)	1 (12.5)	6 (30.0)	12 (7.8)			
	*E. ruminantium*	35 (30.7)				4 (2.6)			
Kajiado County	No. of tick pools	38	11	1		43		4	2
	*Theileria* sp.	1 (2.6)							
	*T. velifera*	2 (5.3)							
	*H. canis*					2 (4.7)			
	*R. africae*	11 (28.9)				1 (2.3)			
	*R. aeschlimannii*		6 (54.5)						
	*Ehrlichia* sp.		1 (9.1)			7 (16.3)		1 (25)	
	*E. ruminantium*	5 (13.2)							

*Am. Amblyomma*, *Hy.* *Hyalomma*, *Rh.* *Rhipicephalus*, *E.* *Ehrlichia*, *R.* *Rickettsia*, *H.* *Hepatozoon*, *T.* *Theileria*, *B.* *Babesia*, *A.* *Anaplasma*, *C.* *Coxiella*

We detected *E. ruminantium* in *Am. gemma* and *Rh. pulchellus* and an *Ehrlichia* sp. in *Hy. rufipes*, *Hy. truncatum*, *Hy. impletatum*, *Rh. pulchellus* and *Rh. evertsi*. Gene sequencing and phylogenetic analysis confirmed three *E. ruminantium* sequences (PV133711-PV133713) shared 100% similarity with *E. ruminantium* strain Welgevonden (CP0630454), while another three (PV133714-PV133716) sequences shared 100% similarity with *E. ruminantium* strain Kumm2 (CP033456) (Fig. 4B). The identified *Ehrlichia* sp. (PV133708-PV133710) shared 100% similarity with *Ehrlichia* sp. sequences from China (OR8325912 and MH974796) and Pakistan (MN726921).

The *R. africae* (PV208561-PV208566) detected in *Am. gemma* and *Rh. pulchellus* shared 100% similarity with reference sequences of *R. africae* from Kenya (MT900495 and MW248723), and *R. aeschlimannii* (PV208553-PV208559) detected in *Hy. rufipes*, *Hy. truncatum* and *Hy. impeltatum* shared 100% similarity with *R. aeschlimannii* reference sequences from Kenya (OR734631 and MT900491).

Two *Theileria* spp., *T. velifera*, and *T. ovis*, and a *Theileria* sp. that could not be resolved to species level were also detected in the tested tick pools (Table 3; Fig. 2; Fig. 4). *Theileria velifera* (PV137961- PV137962, PV137964), which shared 98–100% similarity with sequences from Kenya (MT459435 and PP800988) and Uganda (KU206304), was detected in *Am. gemma*; *T. ovis*, which shared 100% similarity with MW467548, was detected in *Hy. rufipes* and *Rh. pulchellus*; and a *Theileria* sp. (PV137967), which share 98% similarity with reference sequences from Kenya (JQ861975-6) in *Am. gemma* tick pool. In *Rh. pulchellus* tick pools, we also detected *Hepatozoon canis* (PV137954-55) that shared 98% similarity with a reference sequence from Algeria (MK645963). The only *Anaplasma* sp. sequence (PV133720) detected in tick pools was identical to *Am. marginale* detected in blood and shared 100% similarity with references sequences from Kenya (MN266931 and MN266934) and Uganda (KU686782) (Table 3; Fig. 2A-D).

### Tick-borne pathogens in skin swabs

**Fig. 3:**
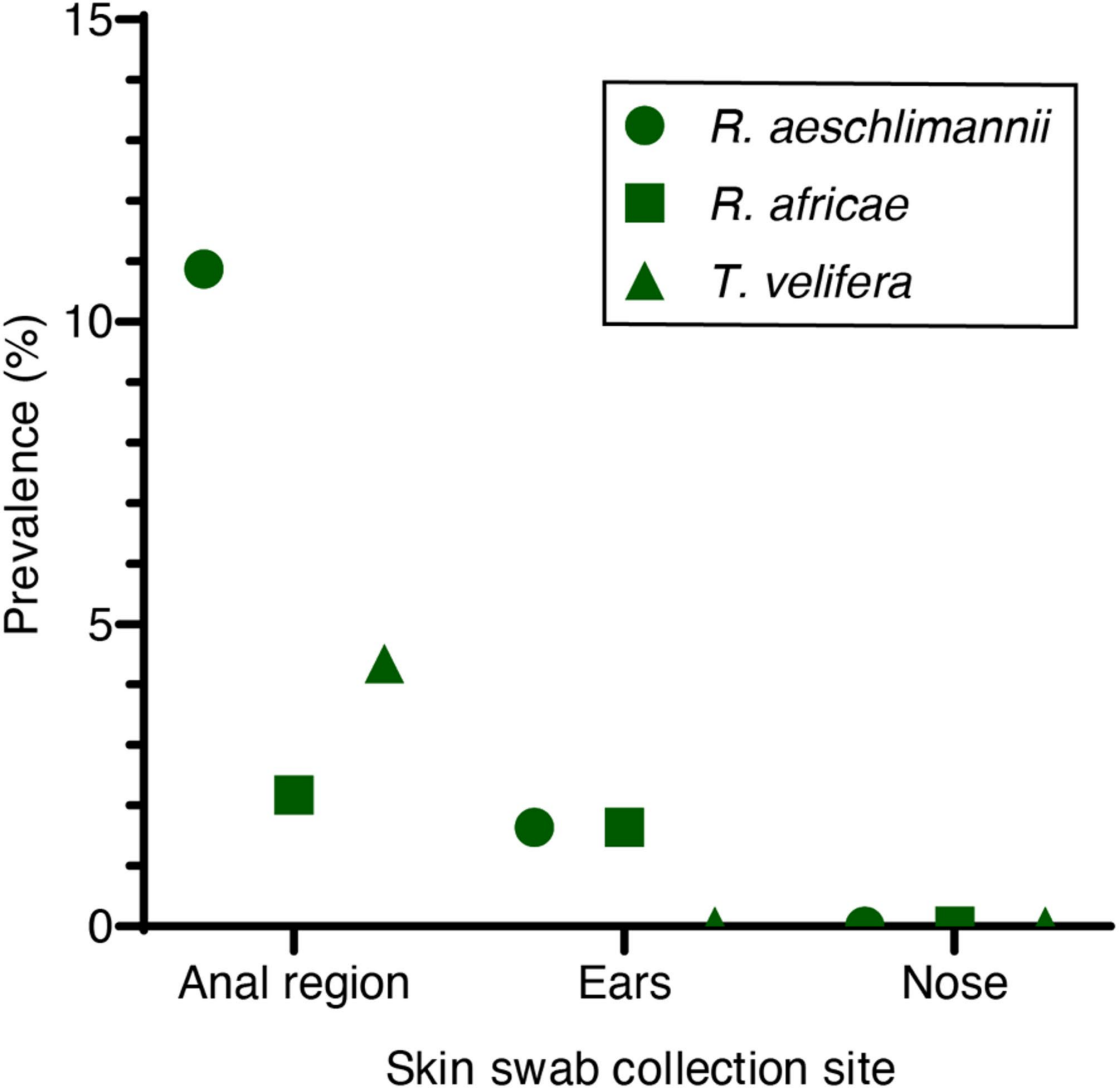
Prevalence of tick-borne pathogens based on cattle skin swab collection site. *R.*= *Rickettsia*;*T.*= *Theileria*

**Fig. 4:**
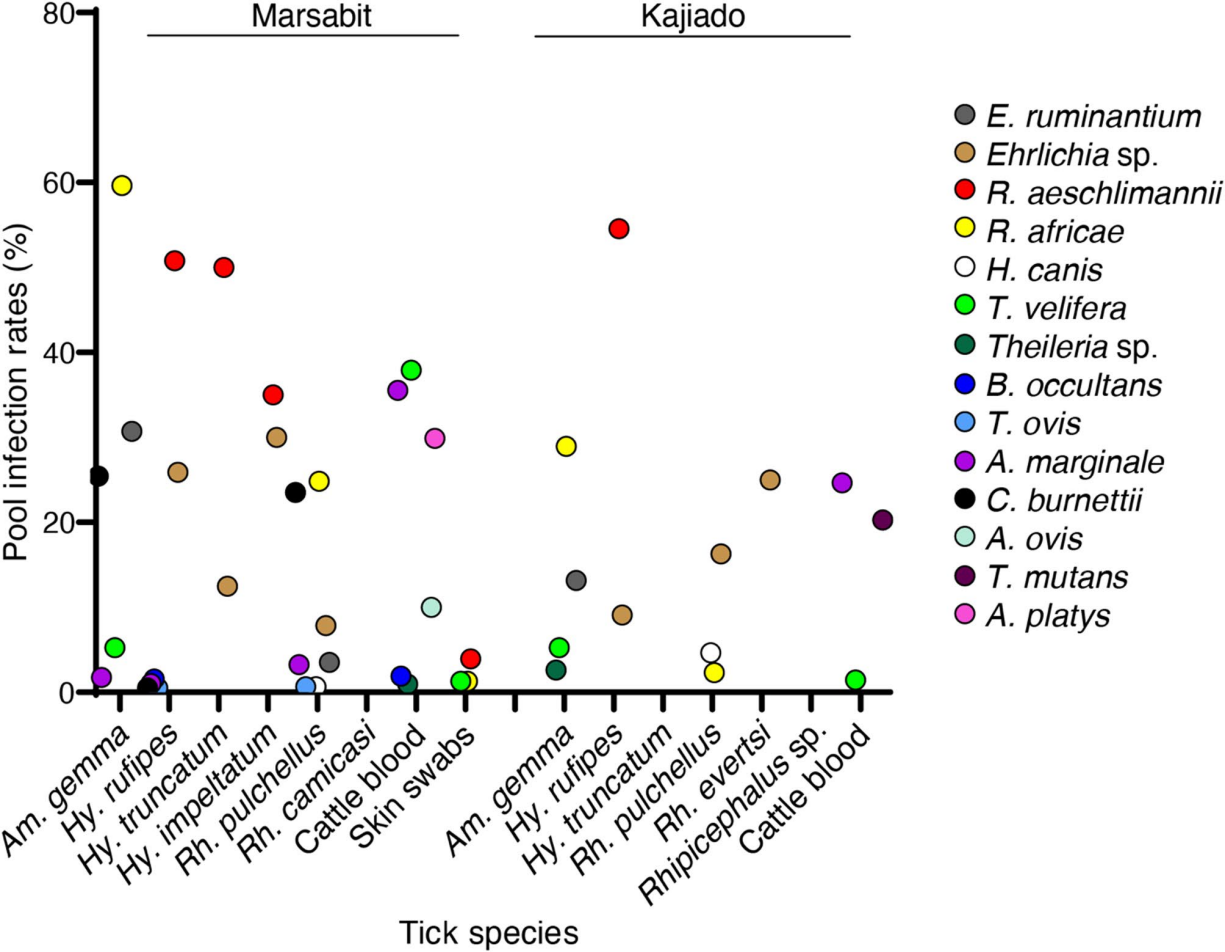
Pathogens detected in blood, ticks, and skin swabs by polymerase chain reaction high resolution melting (PCR-HRM) and their identity confirmed through gene sequencing. *Am.* =*Amblyomma*; *Hy.* = *Hyalomma*; *Rh.* = *Rhipicephalus*; *E.* = *Ehrlichia*; *R.* = *Rickettsia*; *H.* = *Hepatozoon*; *T.* = *Theileria*; *B.* = *Babesia*; *A.* = *Anaplasma*; *C.* = *Coxiella*

A total of 284 skin swabs (153 in Marsabit and 131 in Kajiado) were screened for presence of TBPs by PCR-HRM. We detected *R. africae* and *R. aeschlimannii* in ear and anal swabs and *T. velifera* around the anal region (Fig. 3). *Rickettsia africae*, *R. aeschlimannii*, and *T. velifera* obtained from the skin swabs were identical to the sequences from ticks and shared 100% similarity with reference GenBank sequences from Kenya; *R. africae* sequence (PV208560) shared 100% similarity with MW248723, *R. aeschlimannii* (PV208552) share 100% sequence similarity with sequence MT900493, and *T. velifera* (PV137966) shared 100% similarity with PP800988 (Fig. 2A, C). We did not detect any pathogens in the nose swabs, which is not known to harbour any ticks in cattle. *Rickettsia africae*, *R. aeschlimannii*, and *T. velifera* were the only pathogens detected both in blood and/or ticks and in skin swabs (Tables 3 and 4; Fig. 4).

**Table 4:** Prevalence of tick-borne pathogens in cattle blood and skin swabs in Kajiado and Marsabit counties

Tick-borne pathogens	Prevalence per County					
	Marsabit			Kajiado		
	Blood only	Skin swab only	Both blood and skin swab	Blood only	Skin swab only	Both blood and skin swab
	*n* = 211	*n* = 153 (anal region = 60, ear = 60, nose = 33)	*n* = 60	*n* = 69	*n* = 131 (anal region = 53, ear = 53, nose = 25)	*n* = 53
*A. marginale*	75 (35.5%)			17 (24.6%)		
*A. ovis*	21 (10.0%)			0 (0%)		
*A. platys*	63 (29.9%)			0 (0%)		
*B. occultans*	4 (1.9%)			0 (0%)		
*Theileria* sp.	2 (0.9%)			0 (0%)		
*T. mutans*	0 (0%)			14 (20.3%)		
*T. velifera*	80 (37.9%)	2 (1.3%)	2 (3.3%)	1 (1.4%)		
*R. africae*		2 (1.3%)				
*R. aeschlimannii*		6 (3.9%)				

*A.* *Anaplasma*, *B*. *Babesia*, *T.* *Theileria*

### Co-infection patterns in bovine blood

**Fig. 5:**
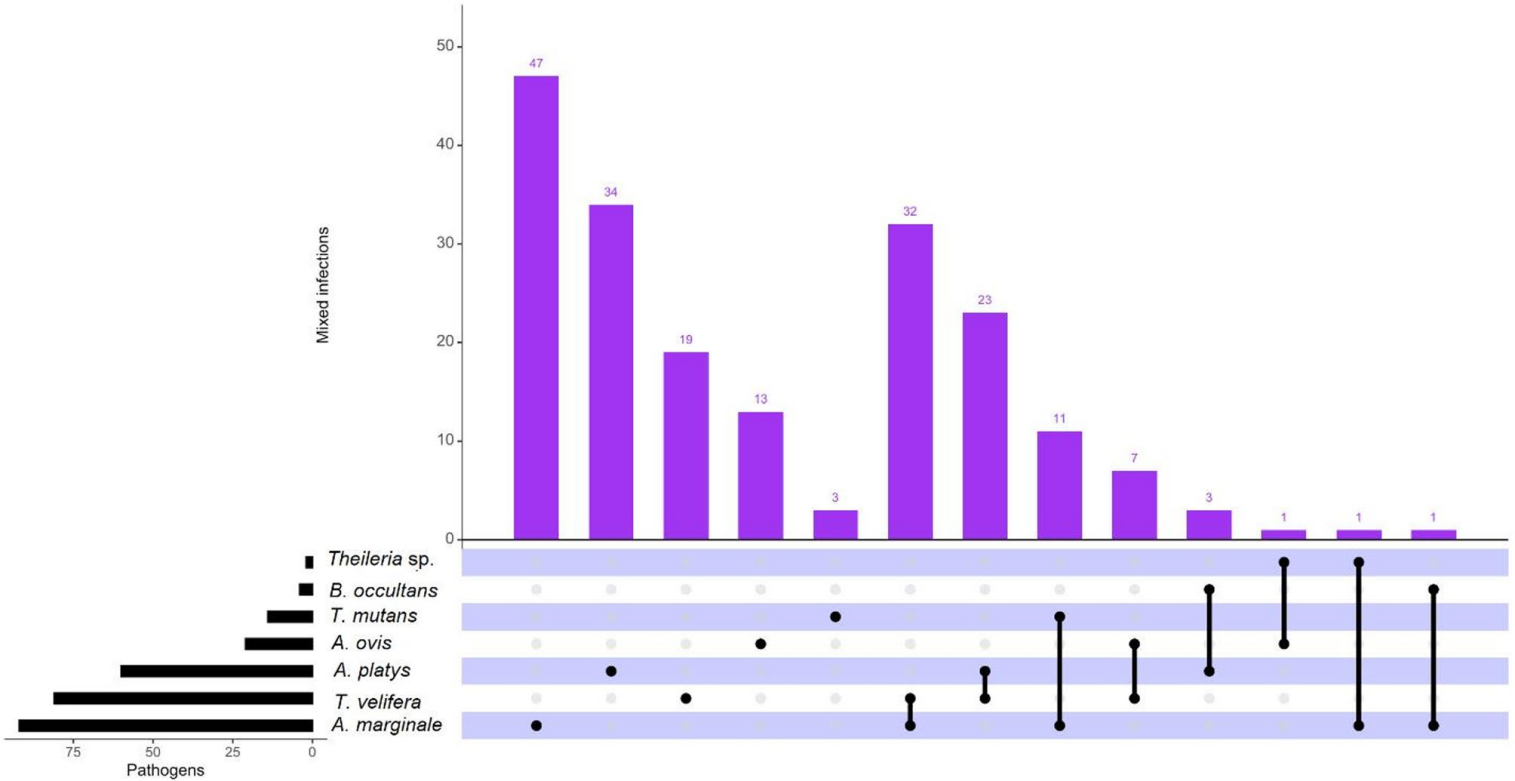
UpSetR plot showing the frequency of mixed infections of TBPs detected in bovine blood in Kajiado and Marsabit counties. The horizontal bars (black bar plot) with labels at the lower left of the panel represent the pathogens detected, with the length of each bar showing the total number of pathogens detected. The matrix with black dots shows single and mixed infections (black dots connected by black lines). The vertical bars at the top of the plot (purple bar plot) show the frequencies of single and mixed infections

The overall TBPs prevalence in cattle across the two counties was highest with *A. marginale* (32.9%), followed by *T. velifera* (28.9%), *A. platys* (22.5%), *(A) ovis* (7.5%), *T. mutans* (5.0%), *B. occultans* (1.4%), and *Theileria* sp. (0.7%) (Table 4; Fig. 5). A total of 115 cattle (41.1%; *n* = 115/280) had a single infection, while 78 cattle (27.9%; *n* = 78/280) were co-infected with two pathogens. *Anaplasma marginale* and *T. velifera* (11.4%; *n* = 32/280) were the most frequent dual infection, followed by a combination of *T. velifera* and *A. platys* (8.2%; *n* = 23/280), *A. marginale* and *T. mutans* (3.9%; *n* = 11/280), and *T. velifera* and *A. ovis* (2.5%; *n* = 7/280). *Anaplasma platys* and *B. occultans* (0.4%; *n* = 1/280), *A. ovis* and *Theileria* sp. (0.4%; *n* = 1/280), *A. marginale* and *Theileria* sp. (0.4%; *n* = 1/280), and *A. marginale* and *B. occultans* (0.4%; *n* = 1/280) were the least common combinations (Table 4; Fig. 5). No triple infections were observed in this study.

## Discussion

Our study characterized TBP infections in blood, ticks, and skin swabs of cattle from Kajiado and Marsabit counties in southern and northern Kenya, respectively. Notably, we documented the presence of multiple TBPs, including the zoonotic *R. africae* and *R. aeschlimannii* in both cattle ticks and skin swabs, and confirmed two strains of *E. ruminantium* (Welgevonden and Kumm2) circulating in ticks from northern Kenya. All ticks collected belonged to three genera (*Amblyomma*, *Rhipicephalus*, and *Hyalomma*), consistent with previous findings in Kenya [12, 14, 24, 35, 40, 41]. Notably, our data also provide updated insights on the local distribution of *Hy. rufipes* and *R. pulchellus* in arid and semi-arid rangelands, enhancing the acarological baseline for these counties. The high prevalence of ticks carrying zoonotic pathogens, combined with frequent contact between livestock and humans, creates significant ongoing health risks for pastoralists, herdsmen, and abattoir workers in southern and northern Kenya. Environmental factors such as changing land use, climate variability, and wildlife–livestock interfaces likely influence tick distribution and pathogen spillover risk.

Q fever, caused by *C. burnetii*, is an endemic zoonosis in Africa [42], especially in resource-limited rural areas where sheep, goats, and cattle serve as major reservoirs for human infection [43]. We observed high prevalence of *C. burnetii* DNA in *Am. gemma* (24.5%) and *Rh. pulchellus* (23.5%) than in *Hy. rufipes* (0.5%) tick pools collected from cattle in northern Kenya, with no detection in ticks from southern Kenya. This exclusive detection in northern Kenya extends prior knowledge on the spatial distribution of *C. burnetii* and suggests the importance of regional control measures. These findings suggest that *C. burnetii* is established in northern Kenya, where *Rhipicephalus* and *Amblyomma* ticks may act as essential enzootic drivers. Our findings corroborate previous studies that found high prevalence of *C. burnetii* in *Rhipicephalus* and *Amblyomma* spp. in Kenya [25, 44, 45], Uganda [46] and Ethiopia [47]. *Coxiella burnetii* DNA has previously been detected in blood and ticks from camels [24, 41, 48], cattle, wildlife, and vegetation [25, 49], with significant seropositivity reported in humans and livestock [43, 48, 50, 51]. Although more than 40 tick species have been found to harbour *C. burnetii* [52], aerosolization of contaminated dust from animal parturition or hide processing poses the primary route for Q fever outbreaks among herdsmen and abattoir workers, which presents *C. burnetii* as a potential biological threat [53]. Nevertheless, the uneven distribution of *C. burnetii* across different regions in Kenyan ticks highlights how Q fever patterns vary by location and time. Understanding these geographic differences is crucial for developing targeted public health strategies and control measures for high-risk areas. More comprehensive studies of *C. burnetii* are needed throughout Kenya to determine how this pathogen affects both animals and humans, and to identify the factors that increase infection risk.

Heartwater, caused by *E. ruminantium* and mainly transmitted by *Amblyomma* spp. ticks, is on the list notifiable diseases of the World Organization for Animal Health (WOAH) due to its economic impact on rural livelihoods in sub-Saharan Africa and the Caribbean and the Indian Ocean islands [54]. We detected *E. ruminantium* at relatively higher rates in *Am. gemma* (30.7), indicating a potential risk for cattle in Kenya. We also detected *E. ruminantium* in *Rh. pulchellus* ticks, an observation like previous studies that confirmed circulation of the pathogen in *Rh. pulchellus* ticks [24, 26, 35, 55, 56]. This finding broadens the recognized vector range for heartwater beyond *Amblyomma* spp. alone, reaffirming earlier suggestions that multiple tick genera may transmit *E. ruminantium* [57–59]. We did not detect *E. ruminantium* in the blood of animals from which positive ticks were collected. However, this is not surprising as *E. ruminantium* primarily infects the endothelial cells of its animal host and has rarely been detected in the bloodstream in the absence of clinical heartwater [60, 61]. Additionally, we detected an *Ehrlichia* sp., closely related to *Ehrlichia* sp. strain WHBMXZ (China) and *Ehrlichia* sp. strain OKARA (Pakistan), in *Hy. rufipes*, *Hy. truncatum*, *Hy. impeltatum*, *Rh. pulchellus* and *Rh. evertsi* tick pools, indicating a potentially broader diversity of *Ehrlichia* species in Kenya. Phylogenetic analysis based on the *Ehrlichia* 16S rRNA revealed close clustering of the *E. ruminantium* sequences in this study with *E. ruminantium* Welgevonden and *E.* Kumm2 strains, underscoring the genetic diversity of this pathogen in the region. Documenting the co-circulation of these specific strains in Kenyan ticks adds to the limited molecular data on *E. ruminantium*, highlighting the need for continued surveillance at the genotypic level. Since some *E. ruminantium* strains may exhibit elevated virulence or zoonotic potential [62], further genomic and epidemiological investigations are warranted to guide effective control measures and mitigate risks to livestock and public health.

We detected *R. africae*, the etiologic agent of African tick-bite fever (ATBF) in humans [63], in high rates (59.6% in Marsabit and 28.9% in Kajiado) in *Am. gemma* tick pools. This concurs with previous studies that found higher rates of *R. africae* in *Amblyomma* spp. from cattle [14, 64]. Although *Amblyomma* ticks were historically considered the sole reservoir for rickettsial infections in sub-Saharan Africa [63], our detection of these pathogens in *Rh. pulchellus* ticks is not surprising, as rickettsial pathogens have been previously detected in *Rhipicephalus* spp [65]. and *Hyalomma* spp [35, 56]. Although *R. africae* infection is common in Africa, clinical cases of ATBF in indigenous people remain underreported, whereas travellers returning from Africa frequently present with this febrile illness [66, 67]. We also detected *R. aeschlimannii* in *Hy. rufipes*, *Hy. truncatum*, and *Hy. impeltatum* tick pools, which corroborates findings from previous studies in Kenya that have reported presence of the pathogen in *Hyalomma* spp. ticks [24, 35, 45]. Notably, none of the bovine blood samples tested positive for *Rickettsia* spp., emphasizing that ticks, rather than cattle bloodstream infections, may be the primary reservoir. Presence of *R. africae* and *R. aeschlimannii* in ticks collected from cattle in northern and southern Kenya highlights the potential risk of infection and illness among the community and animal handlers who are in regular contact with their animals, and among travellers visiting the regions. Our findings also reinforce the importance of including multiple tick genera in rickettsial surveillance to capture the full range of potential vectors.

Additionally, we detected for the first time *R. africae* (1.3%) and *R. aeschlimannii* (3.9%) in skin swabs taken from the ears and anal region of cattle in Kenya. The use of cutaneous swabs for detecting rickettsial pathogens is emerging as a valuable non-invasive diagnostic approach, especially when traditional biopsy methods are impractical. Although *Rickettsia* spp. have also been identified from skin swabs in humans and animals [68–70], our findings did not reveal these pathogens in the corresponding blood samples from swabbed cattle. This discrepancy suggests a localized or transient presence of *Rickettsia* on the skin, under which *Rickettsia* is known to be concentrated. It could have been detected due to release from the skin lesions at tick bite sites or due to tick feces or pathogen deposition at bite sites. Consequently, while skin swabs could supplement standard screening protocols, further validation is essential to ascertain whether positive detection reflects active infection or mere surface contamination. Nevertheless, this approach demonstrates the feasibility of non-invasive sampling for rickettsial pathogens under field conditions, offering a potential adjunct to more conventional methods.

*Anaplasma* spp. affect a wide range of hosts from various taxa and cause considerable losses in livestock. Our study revealed high prevalence of *A. marginale*, *A. platys*, and *A. ovis* in bovine blood, with a lower detection rate of *A. marginale* in ticks removed from the cattle. Notably, *A. marginale*, the cause of gall sickness in cattle, was detected in two *Am. gemma* pools, two *Hy. rufipes* pools, and five *Rh. pulchellus* pools, corroborating previous reports of its widespread distribution in Kenya [13, 14]. Though *A. platys* has always been known as dog pathogen, recent reports in ruminants in Kenya [13, 71], Algeria [72], Senegal [73], and Tunisia [74], as well as in humans [75], points to an expanding host range and emerging zoonotic risk, especially to cattle owners who are in constant contact with their cattle. Given that we observed presence of dogs owned by cattle farmers in the pastoral setting, it is plausible interspecies transmission contributes to *A. platys* circulation. Our findings further underscore that *A. platys* may be adapting beyond its canine host range, illustrating the dynamic nature of *Anaplasma* transmission in pastoral environments. The emergence of *A. platys* as an identified pathogen in dairy cattle underscores a shifting epidemiological landscape in which canine-associated pathogens may be adapting to new hosts or spreading due to environmental or management factors. Such developments highlight the necessity for integrated, multispecies surveillance efforts and sustained tick-control strategies within a One Health framework.

Protozoal parasites such as *Theileria* and *Babesia* are major impediments to livestock production in Kenya and SSA, as they not only lead to economic losses, but also hinder the overall well-being of animal populations [76, 77]. We detected *T. mutans* in bovine blood, mainly in Kajiado County, southern Kenya and *T. velifera*, *Theileria* sp., and *B. occultans* in bovine blood from Marsabit, northern Kenya. We also observed low prevalence of *T. velifera* in skin swabs collected around the anal region. *Theileria velifera* and *T. mutans*, transmitted by several *Amblyomma* tick species and responsible for benign infections, are the most reported species of *Theileria* [14, 64, 78–80]. Although *T. mutans* is known to cause a benign form of theileriosis, pathogenic strains transmitted by *Amblyomma variegatum* have been reported in Narok County, Kenya [81, 82]. The high rate of *T. velifera* in bovine blood in our study is consistent with a study from western Kenya that reported similar findings in cattle [83]. In ticks, we detected *T. velifera* in six pools of *Am. gemma*, and *T. ovis* in one *Hy. rufipes* and one *Rh. pulchellus*, suggesting a diverse range of protozoal parasites infecting cattle in northern and southern Kenya. Identifying *T. ovis* in these tick species broadens our understanding of protozoal parasite transmission routes within the region, emphasizing that multiple tick genera may contribute to sheep and cattle infections. Given the capacity of these pathogens to evolve or increase in virulence, there is need for regular surveillance to monitor the emergence and prevalence of these pathogens in Kenya and prevent possible outbreaks or productivity losses in livestock. Furthermore, cross-sectoral collaboration among veterinary services, public health authorities, and wildlife management departments in both Marsabit and Kajiado counties is essential to translate these findings into coordinated policy and on‐the‐ground interventions. By integrating these efforts into a One Health framework, stakeholders can better coordinate interventions aimed at reducing the burden of tick-borne pathogens on animal and human health and mitigating potential spillover risks to humans.

## Conclusions

This study reports for the first time the co-circulation of two *E. ruminantium* strains (Welgevonden and Kumm2) in ticks and the presence of *R. africae*, *R. aeschlimannii*, and *T. velifera* at tick bite sites in cattle from northern (Marsabit) and southern (Kajiado), Kenya. We report presence of multiple pathogens of both veterinary and public health importance, including zoonotic TBPs such as *C. burnetii*, *R. africae*, and *R. aeschlimannii*, as well as *E. ruminantium* strains capable of causing significant losses in livestock. In addition, we identified *Anaplasma*, *Theileria*, and *Babesia* species circulating in cattle. Although skin swabs showed lower diagnostic yield, the detection of *R. africae*, *R. aeschlimannii*, and *T. velifera* at tick bite sites indicates potential utility for non-invasive surveillance in pastoral systems, pending further validation. To strengthen integrated tick control, the use of faster, cost-effective molecular surveillance methods under a One Health framework could enhance both the detection of TBPs and the rapid implementation of control measures, linking human, animal, and environmental health perspectives, thereby improving disease management strategies. Future research should also aim to unravel the intricate relationships between the identified TBPs and the tick microbiome. This could provide insights into the broader tick-pathogen dynamics and aid in refining strategies to assess and control the TBD risk. By embracing a One Health approach and leveraging robust acarological insights, these efforts will help refine strategies for assessing and controlling TBD risk, ultimately improving disease management and livestock productivity in Kenya’s arid and semi-arid regions.

## Data Availability

All nucleotide sequences obtained in this study have been deposited in the GenBank database under accessions PV133708-PV133730, PV137954-PV137967, PV208552-PV208571.
